# A Re-emerging Respiratory Virus: Human Metapneumovirus (hMPV)

**DOI:** 10.7759/cureus.78354

**Published:** 2025-02-01

**Authors:** Baijayantimala Mishra, Diksha Mohapatra, Manisha Tripathy, Prabhudutta Mamidi, Prasanta R Mohapatra

**Affiliations:** 1 Microbiology, All India Institute of Medical Sciences, Bhubaneswar, Bhubaneswar, IND; 2 Pulmonary Medicine and Critical Care, All India Institute of Medical Sciences, Bhubaneswar, Bhubaneswar, IND

**Keywords:** epidemiology, human metapneumovirus, outbreak, respiratory tract infections, virus

## Abstract

Human metapneumovirus (hMPV) is identified as a pathogenic agent responsible for respiratory tract infections in paediatric, adult and elderly populations. It is a spherical, enveloped virus with a diameter of 209nm, consisting of a single-stranded, non-segmented, and negative-sense RNA genome of around 13.3 kb in length. hMPV infection is prevalent all around the globe, with peak positivity rates detected mostly during later winter and spring seasons. Mostly transmitted through droplet or aerosol contamination, this viral infection may manifest clinical characteristics indicative of both upper and lower respiratory tract infections like fever, cough, rhinorrhea, pneumonia, bronchiolitis, and croup. The recommended laboratory diagnostic approach is reverse transcription polymerase chain reaction, given the challenges associated with culturing the virus. This review article focuses on the structure, replication, genotype, epidemiology, seasonality, transmission methods, clinical manifestations in humans, treatment methodology, and outbreaks of hMPV that have been reported worldwide.

## Introduction and background

Human metapneumovirus (hMPV) is classified as a negative-sense, single-stranded RNA virus which is a common aetiological agent responsible for respiratory tract infection. hMPV was first discovered in the Netherlands in the year 2001, in a monkey kidney cell line while isolating viral pathogens from respiratory samples in 28 epidemiologically unrelated infected children [1]. The cytopathic effect produced was similar to respiratory syncytial virus (RSV), whereas on performing nucleotide sequencing, close similarities were found towards avian metapneumovirus (aMPV). Post discovery the new virus was initially introduced by the International Committee on Taxonomy of Viruses (ICTV) to the subfamily Pneumovirinae within the family Paramyxoviridae. At that time aMPV was the sole member of this genus; hence the newly discovered virus was named hMPV provisionally. Later in the year 2016, this taxonomic assessment was revised and hMPV and aMPV were reclassified as members of the genus Metapneumovirus in the Pneumoviridae family within the order Mononegavirales [2,3].

Worldwide distribution of hMPV has been recorded with seasonal peak in infection noticed during peak winter seasons. The virus is propagated from one individual to another primarily through aerosol transmission or by direct contact with an infected individual with an incubation period ranging from three to five days. Post transmission, the virus is inoculated in the nasopharyngeal mucous membrane following which it spreads rapidly into the respiratory tract infecting the host cells. Engagement of monocytes and lymphocytes within the host endothelium to combat hMPV spread and infection leads to pulmonary inflammation [4,5]. The initial manifestation of hMPV infection typically manifests as mild upper respiratory tract illness with symptoms ranging from fever, malaise, and vomiting to severe clinical features like wheezing, pneumonia, croup, bronchiolitis and uncommon features like conjunctivitis, otitis media, diarrhoea, rash, seizures, and altered liver function tests following hMPV infection [6,7]. The main treatment procedure involves supportive care involving oxygen supplementation, antipyretic and anti-inflammatory medication along with fluid supplementation if necessary.

## Review

Structure

hMPV is characterized as a spherical enveloped virus with a diameter measuring approximately 209 nanometers. hMPV consists of a single-stranded, non-segmented and negative-sense RNA genome of around 13.3 kb in length [8,9]. The viral genome encodes nine structural proteins with varying functions in the following order: 3′-N-P-M-F-M2(-1/-2)-SH-G-L-5′ [8]. The nucleoprotein (N), which possesses a molecular weight of 43.5 kDa, is responsible for the encapsidation and safeguarding of the genomic single-stranded RNA through its binding activity [10]. The phosphoprotein (P), characterized by a molecular weight of 32.4 kDa, functions as a crucial co-factor for the L protein. Its role is vital for ensuring the stability and effective synthesis of new genetic material during its interaction with the RNA-N protein complex [11]. The inclusion bodies commonly observed during hMPV infections predominantly consist of the N and P components [12]. The matrix protein (M) plays a crucial role in the assembly of the newly synthesized virus particle and its subsequent release from the host cell through budding. It features a high-affinity binding site for Ca2+ and serves as the primary component of the virus [13]. The fusion protein (F), possessing a molecular weight of 58.4 kDa, is essential for facilitating the attachment of the virus to the host cell, as well as for the subsequent process of membrane fusion [14]. It is essential to emphasize that the gene associated with the M2 protein consists of two open reading frames (ORFs), which facilitate the expression of either the M2-1 or M2-2 protein. The M2-(1/2) proteins, with molecular weights of 21.2 kDa and 8.1 kDa respectively, are integral to modulating the activity of RNA polymerase and also play a significant role in regulating the immune response initiated by the host [8]. The small hydrophobic protein (SH), with a molecular weight of 20.9 kDa, has multiple roles in modulating the innate immune response, including the inhibition of the interferon (IFN) response, and functioning as a viroporin [15]. The glycoprotein (G), with a molecular weight of 25.7 kDa, interacts with cellular glycosaminoglycans, facilitating the attachment of viral particles to the host cells [16]. Furthermore, substantial evidence suggests that the G protein plays a role in inhibiting the IFN-I response and facilitates the recruitment of neutrophils within the airways by enhancing the secretion of various chemotactic factors, including CXCL2, CCL3, CCL4, IL-17, and tumor-necrosis factor-alpha (TNF-a) [17,18]. The large polymerase protein (L), which has a molecular weight of 230.6 kDa, contains binding sites for zinc and demonstrates multifunctional catalytic activity. This protein is instrumental in the synthesis of new genetic material, working in conjunction with various cofactors [8,19].

Replication

The viral particles associated with hMPV exhibit a range of morphologies, transitioning from spherical to filamentous forms. Additionally, these particles are encased in a lipid membrane that features protein projections with dimensions of approximately 13 to 17 nanometers [1]. The airway epithelial cells (AECs) serve as the principal target for hMPV infection, wherein the virus binds to these cells via the engagement of its glycoprotein with the heparan sulfate present on the AECs' surface [20]. The subsequent attachment of the virus to the cellular membrane is then accompanied by the interaction of the F protein with an integrin present on the surface of AECs. This critical interaction enhances the fusion process between the virus and the cellular membrane, effectively enabling the introduction of hMPV genetic material into the host cell [21]. Upon the initiation of the process, RNA polymerase facilitates the transformation of viral negative-sense RNA into mono-cistronic positive-sense messenger RNA (mRNA). This transformation allows for the translation of the resulting RNA into viral proteins. The synthesized viral glycoproteins are then transported through the Golgi apparatus to the cellular membrane, where they accumulate in preparation for the assembly of newly synthesized viral particles. When the concentration of viral proteins reaches a predetermined threshold, RNA polymerase initiates the replication of the genome into positive-sense RNA. This positive-sense RNA subsequently serves as a template for the synthesis of new genomic negative-sense RNA, which will be integrated into the newly formed viral particles [20]. Ultimately, the M protein facilitates the assembly of viral particles, enabling their release from the cell surface through the process of budding from the membrane [20].

Genotype

hMPV is classified into two primary genotypes, designated as A and B. Each of these genotypes is further subdivided into two distinct subgroups: A1 and A2 for genotype A, and B1 and B2 for genotype B. Additionally, subgroup A2 is further classified into three more specific categories: A2a, A2b, and A2c [8,22-25]. The hMPV G protein exhibits considerable nucleotide variation, which contributes to the genetic diversity observed across different genotypes. Specifically, the nucleotide conservation of the G gene exhibits differences among genotypes ranging from approximately 45% to 53%, while the conservation of amino acids fluctuates between approximately 22% and 27% [26]. hMPV has a widespread presence, with the A2b sub-lineage of genotype A2 being dominant globally for multiple years [25,27]. It is important to highlight that a duplication region consisting of 111 or 180 nucleotides in the G gene has been identified in the A2b strains [28]. Prior investigations have indicated that hMPV is likely associated with severe illness in children. However, certain studies indicate that the A2c genotype has a higher propensity to lead to disease in younger adults compared to children [29]. The circulating lineages identified in India comprise B1, B2, A2b, and A2c [25,27,30].

Epidemiology

hMPV infection have been reported all around the globe, reportedly in all the continents [26,31-37]. hMPV has the potential to impact individuals across all age demographics; however, it is observed to be more prevalent among children under the age of five. Respiratory tract infections are progressively acknowledged as a substantial contributor to both morbidity and mortality on a worldwide basis. They are currently ranked as the second leading cause of mortality among children under the age of five, irrespective of geographic variations [38]. hMPV represents one of the predominant etiological factors for respiratory tract infections and accounts for approximately 10% of acute hospitalization instances in paediatric patients under the age of five [39]. Similarly, 15% of community-acquired pneumonia cases requiring hospitalisation in children under and above five years of age are also caused by hMPV [40]. A study by Walsh et al. states that hMPV infection is also common in the adult population, but often remains asymptomatic. The prevalence of hMPV infection among adults varied between 3% and 7.1% across four subsequent winter seasons [41]. This outcome aligns with the annual average infection rate of RSV, which stands at 5.5%. However, it is notably higher than the annual average infection rate for influenza, recorded at 2.4% [42]. The rate of hospitalisation of hMPV-associated respiratory tract infection in adults aged 50 years or above is 4.5% while the hospitalisation rates due to RSV or influenza A-associated respiratory tract infection are 6.1% and 6.5% respectively [43].

Primary hMPV infection usually occurs by the age of six months, following which reinfection can occur throughout life [44]. RSV primarily affects premature infants and children under three months of age. In contrast, older children are often diagnosed with hMPV [45]. Evidence suggests that hMPV is associated with substantial cases of lower respiratory tract infection (LRTI) in infants and young children and ranks second following RSV in causing bronchiolitis in children [34,46]. Incidence of hMPV-associated LRTI in young children ranges from 5-15% in different geographical locations in different periods of time, with higher rates being also reported in several other studies [47-50].

Seasonality and transmission

hMPV predominantly circulates during the late winter and spring seasons in the temperate region [51]. Some studies have also found hMPV infection during summer and early autumn [52-54]. Transmission of hMPV primarily occurs through direct or proximate contact with the respiratory secretions of infected individuals which may be saliva, large particles of aerosol or droplets, or by contact with surfaces contaminated by their secretion. Apart from symptomatic individuals, asymptomatic individuals can also be responsible for transmission of hMPV infection with two studies finding a transmission rate of 4.1% amongst hMPV asymptomatic cases [55,56].

Clinical manifestations

Since the year 2001, hMPV has emerged as a principal etiological agent in the progression of respiratory tract infections in both children as well as adult populations. The spectrum of hMPV infection ranges from less severe forms of upper respiratory tract infection (URTI) like fever, cough, and rhinorrhoea to more severe forms of LRTI like wheezing, croup, bronchiolitis and pneumonia [57]. In addition to its association with respiratory illnesses in infants and children, hMPV may also induce respiratory tract infections in immunocompromised individuals and older adults and individuals with underlying medical conditions like chronic obstructive pulmonary disorders (COPD), asthma or cancer [56,58]. In certain aspects, clinical manifestations of hMPV are indistinguishable from clinical manifestations of RSV due to presentation of features like fever, cough, breathlessness, rhinorrhoea and hypoxia. On radiological diagnosis, distinctive features like hyperinflation, presence of infiltrate and peribronchial cuffing can be seen in chest radiographs [59,60].

Researchers have found that the symptoms of hMPV infection seen in older children are similar in characteristics with symptoms seen in children less than five years of age [57]. A significant proportion of adults who tested positive for hMPV demonstrate serological markers indicative of prior infection. However, it is important to note that older adults are at an increased risk of infection due to a diminishing immune response [56]. Studies carried out in Germany and Japan suggest that >90% of the cohort group from 60 to 89 years of age with presence of neutralizing antibodies were infected with hMPV infection, suggesting that a positive antibody titer cannot provide full protection from infection [61,62].

Like other respiratory pathogens, hMPV has also been detected in children with acute otitis media [63-65]. In one study acute otitis media was detected in 50% of patients with hMPV-associated URTI, [66] while in another study concomitant otitis media was diagnosed in one-third of patients with hMPV-associated LRTI [39]. Similarly in one study, hMPV was detected in 6% nasal leverage of children diagnosed with acute otitis media, suggesting that hMPV may be one of the causative agents of otitis media in children along with other pathogens [65].

Several studies have highlighted causative role of hMPV in asthma exacerbations, [67,68] whereas a study by Rawlinson WD et al. did not observe any such association during the course of their research [69]. hMPV has also been detected in adults detected with acute asthma exacerbation requiring hospitalisation [70]. Not only asthma, but hMPV has also been detected in adults diagnosed with pneumonia, influenza-like illness (ILI), bronchiolitis and COPD [71]. The prevalence of hMPV infection is comparatively lower in the adult population than in children, but the fact that hMPV-associated diseases can be detected in all age groups cannot be neglected [56].

Due to its seasonal distribution, hMPV in many instances detected as a co-infection pathogen with RSV. Various studies have found a co-infection rate of <10%, [48,72,73] while only one study has reported a 70% co-infection rate of hMPV with RSV, causing more severe infection as compared to hMPV mono-infection [74].

Diagnosis

Reverse transcription polymerase chain reaction (RT-PCR) represents the predominant methodology employed for the detection of hMPV. Real-time RT-PCR, which is utilized for the detection of various respiratory pathogens, can also be effectively employed for the identification of hMPV. The in vitro replication of hMPV is limited to a select number of cell lines that necessitate trypsin for optimal propagation, resulting in suboptimal growth in cell culture settings. Additionally, hMPV exhibits a slow growth rate when cultivated using traditional cell culture methodologies and demonstrates only mild cytopathic effects [75]. Laboratory detection of hMPV using enzyme-linked immunosorbent assay (ELISA) is not in practice. Immunofluorescence using virus-specific antibodies can also be used for diagnosis of hMPV, though this laboratory diagnostic procedure may not be as sensitive and specific as RT-PCR [1,76].

Vaccines and anti-viral therapy

The current imperative is to develop and administer a safe and effective vaccine to provide protection against hMPV infection. Several vaccine proto-types are currently under trial procedure and being tested in experimental animal models. The hMPV F gene incorporated into live recombinant human parainfluenza virus has shown results of induction of hMPV-specific antibodies in experimental animals providing them protection against hMPV infection [77]. In a supplementary investigation, researchers constructed a chimeric parainfluenza virus Type 3, which integrates both bovine and human components (designated as b/h PIV3). This construct incorporates the F and hemagglutinin-neuraminidase (HN) proteins derived from human parainfluenza type 3 (hPIV3). This innovative methodology facilitates the expression of the F protein of the hMPV from the second genome position (labelled b/h PIV3/hMPV F2), with the primary aim of advancing a vaccine for hMPV. It is noteworthy that this vaccine candidate has exhibited efficacy in providing protection to experimental subjects against hMPV infection [78]. Another method of providing protection against hMPV infection is employing live, recombinant, gene-deleted hMPV as a vaccine based on similar experiments using recombinant viruses for prevention of RSV [79]. Apart from influenza virus, antiviral therapy has not shown any effective management of other respiratory viruses. Ribavirin is a nucleoside compound that exhibits extensive inhibitory activity against a wide range of both DNA and RNA viruses. Ribavirin has produced significant results in inhibition of TNF-a, interleukin-10 and IFN-gamma, terminating T-cell-mediated damage caused by viral pathogens [80,81]. Ribavirin is also classified as a teratogen and administration is required to be carried out by nebulization using a small particle aerosol generator [82]. On the contrary, ribavirin therapy has not shown prominent results in limiting RSV infection, and hence use of ribavirin in managing hMPV remains contentious [83,84].

A recently discovered approach is the use of RNA interference (RNAi) against RNA virus infections. RNAi represents a naturally occurring intracellular mechanism that regulates gene expression through the targeted silencing of specific mRNAs. RNAi as a therapeutic agent has shown substantial results in managing respiratory infections by RSV, influenza and parainfluenza both in vitro and in vivo. Hence more studies are required targeting towards efficiency of RNAi as a therapeutic agent in managing hMPV infections [85,86].

Outbreaks reported

**Figure 1:**
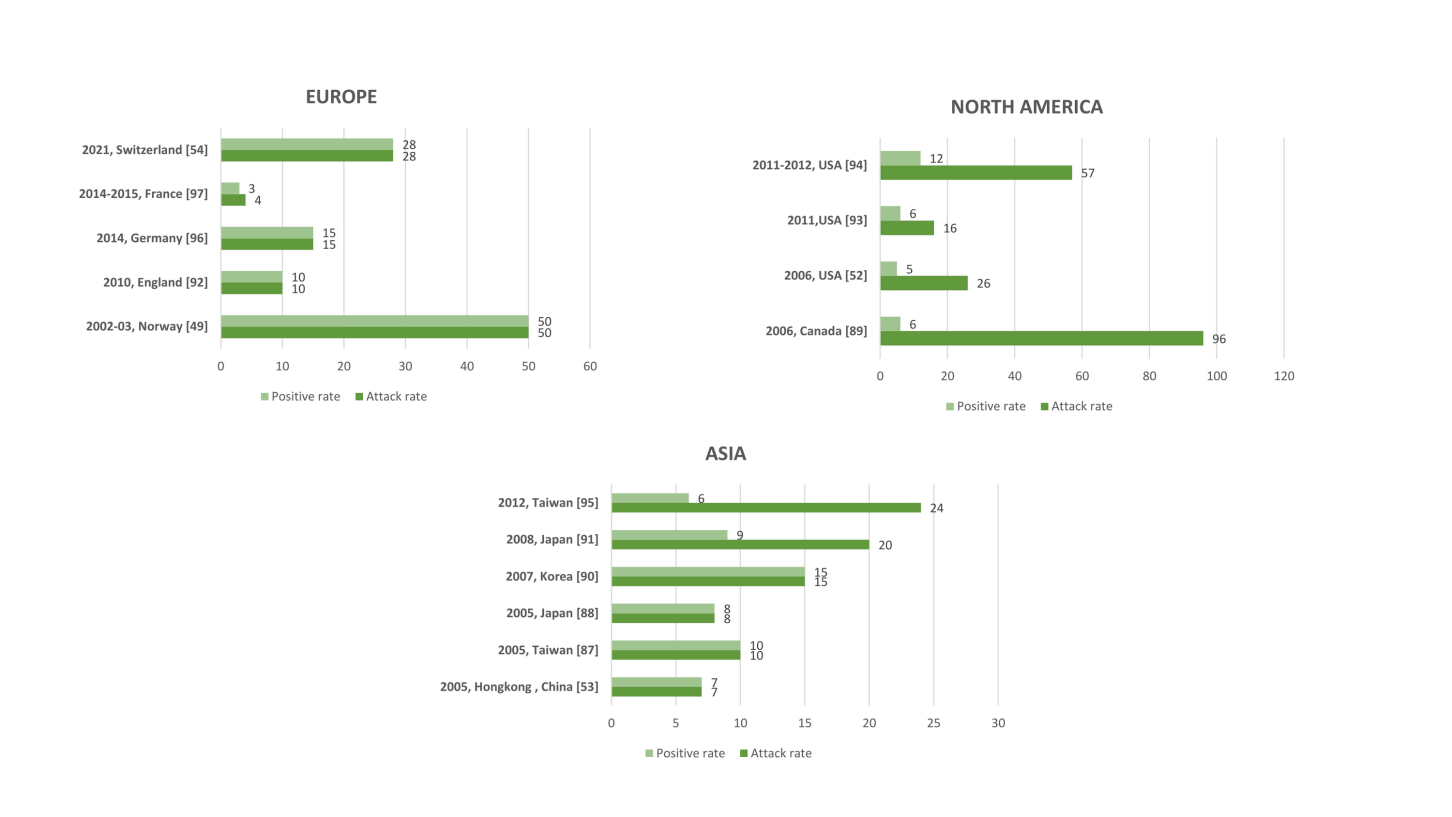
Human metapneumovirus (hMPV) outbreaks reported worldwide from 2001-2024

Since its discovery a total of 15 prospective hMPV outbreaks have been reported worldwide, out of which five outbreaks were in Europe, four in North America and six in Asia, as specified in Figure 1. hMPV outbreaks have been seen not only in children but in young as well as older adults too. Some studies have also reported hMPV outbreaks in off-seasons like summer and early autumn [52-54]. Patients have exhibited clinical signs indicative of both upper and lower respiratory tract infections. Details regarding all the prospective hMPV outbreaks reported worldwide have been illustrated in Table 1.

**Table 1:** Details of human metapneumovirus (hMPV) outbreaks reported worldwide. URTI: upper respiratory tract infection, LRTI: lower respiratory tract infection

Year of outbreak	Place	Population	Total sample Tested	Age group	Attack Rate	Positive Rate	Season	Male/Female Ratio	Type of infection	Death
2002-03 [49]	Norway	Children	236	Median age 12 months	50	50	November-April	32:18	URTI + LRTI	Not specified
2005 [53]	Hongkong, China	Adults	31	Mean age 66	7	7	July-August	Not specified	URTI	Not specified
2005 [87]	Taiwan	Adults	13	Mean age 54.1 years	10	10	May	Not specified	URTI	1 of 10
2005 [88]	Japan	Older Adults	23	Mean age 79 years	8	8	January	Not specified		Not specified
2006 [89]	Canada	Older Adults	364	Mean age 83 years	96	6	January-February	Not specified	URTI + LRTI	3 of 6
2006 [52]	California, USA	Older Adults	148	Mean age 70.2 years Median age 72.5	26	5	June-July	11:15	URTI	Not specified
2007 [90]	Korea	Children	2200	Mean age 1.6 years	15	15	March-May	11:4	LRTI	Not specified
2008 [91]	Japan	Adults	44	Age group: 15-60 years	20	9	April	13:7	URTI + LRTI	Not specified
2010 [92]	England	Older Adults	34	Mean age 85 years	10	10	July-September	Not specified	URTI	1 of 10
2011 [93]	Oregon, USA	Older Adults	44	Not specified	16	6	Late spring-summer	Not specified	URTI	2 of 6
2011-2012 [94]	West Virginia and Idaho, USA	Older Adults	83	Median age 84 years	28	12	December-February	Not specified	URTI + LRTI	1 of 12
2012 [95]	Taiwan	Adults	73	Mean age 42 years	24	6	March-April	34:39	Not specified	Not specified
2014 [96]	Germany	Adults, seniors	Not specified	Not specified	15	15	January- February	Not specified	Not specified	4 of 15
2014-2015 [97]	France	Older Adults	78	Median age 86.5 years	4	3	January	36:42	Not specified	1 of 3
2021 [54]	Switzerland	Adults and Children	Not specified	Age group: Children 0-4yrs Adults 20-90yrs	28	28	June-July	Not specified	URTI + LRTI	Not specified

## Conclusions

hMPV was present earlier and currently, it is a re-emerging respiratory pathogen causing significant morbidity and mortality, particularly among young children, the elderly and immunocompromised individuals. A greater clarity of hMPV prevalence rate, epidemiology, seasonality, and immunologic response towards the infection needs to be elucidated by conducting more studies related to the aspect targeting a larger population. Evolutionary studies suggest that hMPV has been an etiological agent of respiratory infections even years before its first detection. However, enhancing surveillance programs and more robust studies for developing treatment modalities and vaccines against hMPV infection is the need of the time for immediate detection, efficient treatment facilitation and prevention of hMPV infection. 
